# Post-embryonic organogenesis and plant regeneration from tissues: two sides of the same coin?

**DOI:** 10.3389/fpls.2014.00219

**Published:** 2014-05-26

**Authors:** Juan Perianez-Rodriguez, Concepcion Manzano, Miguel A. Moreno-Risueno

**Affiliations:** Department of Biotechnology, Center for Plant Genomics and Biotechnology, Universidad Politecnica de MadridMadrid, Spain

**Keywords:** development, organ formation, pluripotency, callus, auxin, cytokinin, *de novo* organogenesis, cell fate

## Abstract

Plants have extraordinary developmental plasticity as they continuously form organs during post-embryonic development. In addition they may regenerate organs upon *in vitro* hormonal induction. Advances in the field of plant regeneration show that the first steps of *de novo* organogenesis through *in vitro* culture in hormone containing media (via formation of a proliferating mass of cells or callus) require root post-embryonic developmental programs as well as regulators of auxin and cytokinin signaling pathways. We review how hormonal regulation is delivered during lateral root initiation and callus formation. Implications in reprograming, cell fate and pluripotency acquisition are discussed. Finally, we analyze the function of cell cycle regulators and connections with epigenetic regulation. Future work dissecting plant organogenesis driven by both endogenous and exogenous cues (upon hormonal induction) may reveal new paradigms of common regulation.

## INTRODUCTION

Plants can generate organs and tissues throughout their whole life (Birnbaum and Sanchez Alvarado, 2008; Dinneny and Benfey, 2008). Post-embryonic formation of organs initially arises from the shoot and root apical meristems, which are also known as primary meristems. Primary meristems are formed as a result of embryogenesis and upon activation during germination, they start generating main root(s), leaves and flowers (Peris et al., 2010; Besnard et al., 2011; Sozzani and Iyer-Pascuzzi, 2014). These meristems are source of continuous growth but not exclusively, as lateral or secondary meristems are equally important to model plant architecture and determine developmental plasticity upon environmental insult. Plants, as sessile organisms, are often exposed to adverse conditions such as disease and injury by herbivores, hail, lightning, etc. Then, growth and survival relies on production of lateral meristems. Moreover, formation of lateral meristems is sometimes used as reproductive or propagation strategy in some species to generate new individuals, (e.g., production of adventitious roots and shoots in *Cyperus papyrus* or *Rubus fruticosus*). These regeneration capabilities of plants have been exploited in agriculture for propagation purposes of selected varieties, virus sanitization and development of biotechnological tools (Sussex, 2008; Bhatti and Jha, 2010; Taskin et al., 2013). Plant regeneration can be achieved *in vitro* from explants of plant tissue cultured in hormone containing medium. Different ratios of the plant hormones auxin and cytokinin direct the developmental fate of regenerating tissues to form shoots or roots (Skoog and Miller, 1957; Valvekens et al., 1988).

During post-embryonic developmental programs lateral meristems are formed *de novo*, conversely to embryonic primary meristems. This formation requires in many cases reprograming and changes in cell fate (Chandler, 2011). This natural cell reprogramming changes the developmental potential of certain cells conferring them extraordinary unique properties. Recent studies have shown that post-embryonic reprogramming underlies the basis of plant regeneration (Atta et al., 2009; Sugimoto et al., 2010). Comparison of the developmental mechanisms that drive formation of new organs during development and during *de novo* organogenesis (upon exogenous hormonal treatments) revealed a series of common mechanisms and regulators. In this review, we summarize recent advances in the field and discuss parallelisms between both processes.

## FORMATION OF NEW ORGANS DURING PLANT DEVELOPMENT

Formation of post-embryonic organs is associated with changes in cell fate. In the shoot apical meristem, certain cells or clusters of cell exposed to endogenous cues or biomechanical-mediated signals form new organs. These meristematic cells change their developmental program to form the multiple tissues of the new organ. In contrast, neighbor cells which are not exposed to these cues differentiate. Formation of aerial organs in the shoot apical meristem occurs in a predictable pattern known as phyllotaxis. In the model plant *Arabidopsis thaliana*, development of new aerial organs is initiated by the plant hormone auxin (Reinhardt et al., 2000; Hamant et al., 2010; Besnard et al., 2011). Accumulation of auxin in certain cells of the shoot meristem is required to form leaves and flowers (Reinhardt et al., 2003; Heisler et al., 2005; Besnard et al., 2011). However, it has been proposed that organ initiation requires previous specification of founder cells. In support of this hypothesis founder cell specification has been shown to precede establishment of auxin response maxima during flower initiation (Chandler, 2011; Chandler et al., 2011). Auxin maxima are achieved through the formation of local gradients that result of the activity of intercellular auxin transporters, such as PIN-FORMED 1 (PIN1). As PIN1 expression is, in turn, activated by auxin this regulation forms a feedback mechanism that creates auxin maxima at the position where new organs are initiated (Reinhardt et al., 2003; Heisler et al., 2010). Auxin depletion from surrounding cells is believed to inhibit organ formation. In addition, auxin generates downstream inhibitory signaling fields of the hormone cytokinin. Formation of these signaling fields occurs through movement of the cytokinin inhibitor ARABIDOPSIS HISTIDINE PHOSPHOTRANSFER PROTEIN 6. Cytokinin signaling patterns in the shoot meristem are required for initiation of new organs following the temporal sequence typical of phyllotaxis (Besnard et al., 2014). Formation of the aerial system also requires the activity of lateral shoot meristems. Lateral or axillary meristems are derived from the primary shoot apical meristem, although the developmental mechanism has not been described at the molecular level. The axillary meristems have similar potential as the primary meristem and can remain dormant or be activated to produce a branch (Muller and Leyser, 2011).

**FIGURE 1:**
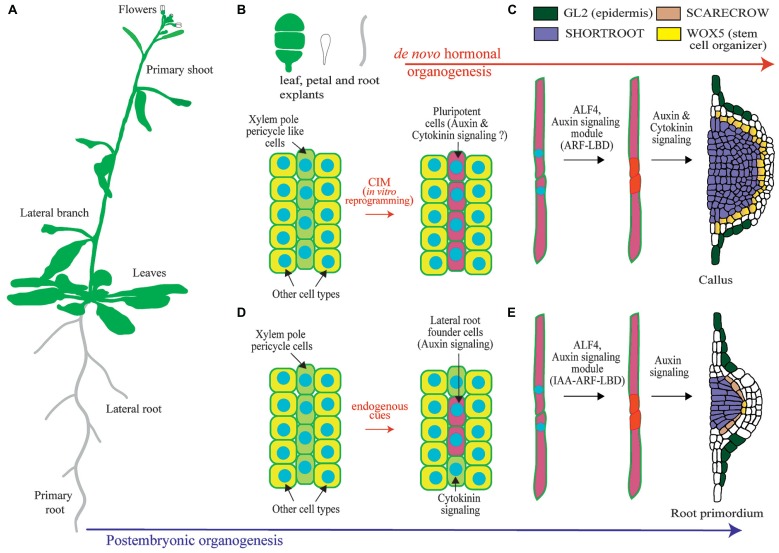
**Overview of lateral root organogenesis in the model plant *Arabidopsis thaliana* and comparison with callus formation. (A)** Schematic of an *Arabidopsis* plant where primary and lateral organs are shown. **(B)** Upon hormonal induction during *in vitro* culture, tissue explants can be reprogrammed. Only xylem-pole-pericycle-like cells are susceptible of developmental reprograming. **(C)** Pluripotent cells divide to form a callus. **(D)** Xylem-pole-pericycle cells are normally reprogramed during post-embryonic development to form lateral root founder cells. Note that as not all cells are reprogrammed this creates a branching pattern. **(E)** Lateral root initiation occurs through the asymmetric cell division regulated by ALF4 and auxin signaling. Subsequent divisions create a primordium with stereotypic morphologies and expression of the root regulators SHORTROOT, SCARECROW, WUSCHEL RELATED HOMEOBOX 5 (WOX5), and GLABRA2 (GL2). Note the similarities with callus formation.

In the root, lateral organs are not originated from the root apical meristem, but from lateral meristems. In *Arabidopsis* new lateral root (LR) meristems are initiated in the differentiation zone of the root from pairs of founder cells that derive from the pericycle (**Figure 1**; Malamy and Benfey, 1997; Lucas et al., 2013). The *Arabidopsis* root is organized in tissues arranged in concentric layers (Dolan et al., 1993). The pericycle surrounds the central vascular cylinder and it is, in turn, surrounded by the ground tissue and the epidermis. Specification of cells to become LR founder cells occurs in pericycle cells adjacent to the xylem (the xylem pole pericycle). However, not all xylem pole pericycle cells are specified as LR founder cells but only specific subsets. It is unknown if these subsets of xylem pole pericycle cells are maintained into an undifferentiated state while they progress through the root developmental zones or if they dedifferentiate and redifferentiate into LR founder cells (Malamy and Benfey, 1997; Dubrovsky et al., 2000). LR founder cells show differential gene expression as compared to pericycle cell types which indicates that they must undergo transcriptional reprogramming as part of their specification (Dubrovsky et al., 2008; Goh et al., 2012a; Manzano et al., 2012). Xylem pole pericycle cells that are not specified as LR founder cells undergo differentiation into pericycle. In contrast to LR founder cells, xylem pole pericycle cells do not normally develop into new organs, and therefore they cannot be considered to be pluripotent or stem cells. However, pericycle cells maintain the capacity of being reprogrammed upon exogenous hormonal treatment or induced biosynthesis of auxin (De Smet et al., 2007a; Dubrovsky et al., 2008; Sugimoto et al., 2010). Specification of LR founder cells during post-embryonic development has been associated to auxin accumulation, as LR founder cells where initially defined as xylem pole pericycle cells showing expression of the auxin transcriptional reporter element DR5 (DIRECT REPEAT5) fused to the green fluorescent protein (GFP; Dubrovsky et al., 2008). Recent evidence indicates that endogenous auxin accumulation is required for LR founder cell activation and division, but it may not be necessary for its specification. LR founder cells show expression of DR5 prior to accumulation of the auxin transporter PIN3 (Marhavy et al., 2013). Specification of LR founder cells could be related to a pre-pattern mechanism known as the LR clock. Cells exposed to in-phase gene expression oscillations of the LR clock form prebranch sites. Prebranch sites are defined by expression of DR5 fused to the luciferase. Thus, they could define LR founder cells or an earlier developmental stage, as the luciferase reporter is more sensitive than the GFP (Van Norman et al., 2013). In agreement with this, loss of function mutants of oscillating transcription factors which are impaired in prebranch formation are also impaired in formation of LRs (Moreno-Risueno et al., 2010, 2012). Subsequent to the specification of pericycle cells as LR founder cells, these will divide asymmetrically. This is the first step of LR initiation and precedes several rounds of divisions which results in the formation of a primordium. This primordium eventually grows through the ground tissue and the epidermis to develop a new LR (Malamy and Benfey, 1997; Lucas et al., 2013).

## THE FIRST STEPS OF *DE NOVO* ORGANOGENESIS UPON HORMONAL INDUCTION REQUIRES CHANGES IN DEVELOPMENTAL POTENTIAL, CELL FATE, AND A ROOT DEVELOPMENTAL PATHWAY

Plant cells have high developmental plasticity and initial cell fate can be entirely changed during post-embryonic development (van den Berg et al., 1995). This developmental plasticity has also been observed upon hormonal treatments. Exogenous auxin application results in the production of new organs: leaves and flowers in the shoot and roots in the underground part of the plant (Reinhardt et al., 2003; De Smet et al., 2007b). Treatment with multiple hormones can be used to sequentially regenerate a whole plant from excised explants and even an embryo can be formed from somatic cells (Su and Zhang, 2014; Xu and Huang, 2014).

Using a combination of treatments with an auxin transport inhibitor and the synthetic auxin analog 1-naphthalene acetic acid, endogenous LR patterning mechanisms can be overridden. The result is the synchronous division of all root xylem pole pericycle cells (Himanen et al., 2002). Interestingly, these divisions follow the same pattern observed during the initiation of LRs and result in the production of primordia that develop along the length of the root following the xylem axis. This method, which was termed the LR inducible system, was used to perform genome-wide approaches. Thereby, transcriptional profiling was used to dissect the molecular mechanism leading to initiation and formation of new LRs. These studies characterized novel proteins involved in post-embryonic LR formation (De Smet et al., 2008; De Rybel et al., 2010) and established connections between auxin and progression through the cell cycle during LR initiation (Himanen et al., 2004; Vanneste et al., 2005). These findings indicate the existence of common regulatory mechanism between endogenous and hormonal-induced organogenesis despite the obvious differences in the distribution of lateral organs.

Further insight into the molecular mechanisms operating during *de novo* organogenesis upon hormonal induction come from studies performed on proliferating masses of cells, which are commonly designed as calli (singular: callus). The term callus had been previously used to designate outgrowth of cells associated with callose accumulation and wounding (Ikeuchi et al., 2013). In this review we will use the term callus to refer to a proliferating mass of cells. Plant explants can be reprogrammed to form callus upon culture in a medium containing auxin and cytokinin, which is known as callus inducing medium (CIM). Because callus can eventually form shoots and roots, and somatic cells can be reprogrammed through hormonal treatments to form embryos, all plant cells have been traditionally considered to be totipotent (Ikeuchi et al., 2013). However, plant cells need to be reprogramed in order to change their developmental programs and undergo organogenesis. Therefore plant cells are not totipotent *per se*, although cell fate appears to be entire regulative. Furthermore, recent studies show that only xylem pole pericycle like cells can be reprogrammed to form callus whereas other tissues do not change their developmental potential when grown on CIM (**Figure 1**; Atta et al., 2009; Sugimoto et al., 2010).

Regenerative characteristics of xylem pole pericycle cells could relate to embryonic properties and subsequent derived callus could follow developmental programs typical of embryogenesis. However, overexpression of embryonic fate regulators in post-embryonic tissues generates masses of cells where embryonic genes are expressed and somatic embryos generated (Boutilier et al., 2002; Tsuwamoto et al., 2010; Koszegi et al., 2011). As CIM treatment does not result in formation of embryos, it is very unlikely that callus derived from xylem pole pericycle cells follows an embryonic developmental program. Likewise, several studies show that calli generated from shoot, petal, or root cells follow a root developmental pathway and are enriched in root-tip expressed genes (Sugimoto et al., 2010). Gene expression and cellular markers are normally used to explore changes in cell fate. Based on the expression patterns of regulators of shoot and root developmental programs or those of cell type specific reporters, calli were shown not to be the undifferentiated structures widely believed. Calli derived from aerial and root organs showed an organized pattern where the main root tissues were present. Strikingly these tissues were arranged following the organization of a root meristem (**Figure 1**; Atta et al., 2009; Sugimoto et al., 2010). Further insight into the developmental mechanism involved in callus formation comes from the analysis of the regulator ABERRANT LATERAL ROOT FORMATION 4 (ALF4). ALF4 is required for the first asymmetric division of xylem pole pericycle cells during LR initiation. In addition, it is also required for callus formation (Sugimoto et al., 2010). Based on this finding and the fact that callus is specifically formed from xylem pole pericycle cells, it appears that the initial steps of plant regeneration from explants are very likely under the same genetic program as LR initiation. In this scenario, specification of LR founder cell like cells could also occur.

## AUXIN AND CYTOKININ ARE CENTRAL ENDOGENOUS SIGNALING MOLECULES THAT REGULATE LATERAL ROOT INITIATION AND CALLUS FORMATION

**FIGURE 2:**
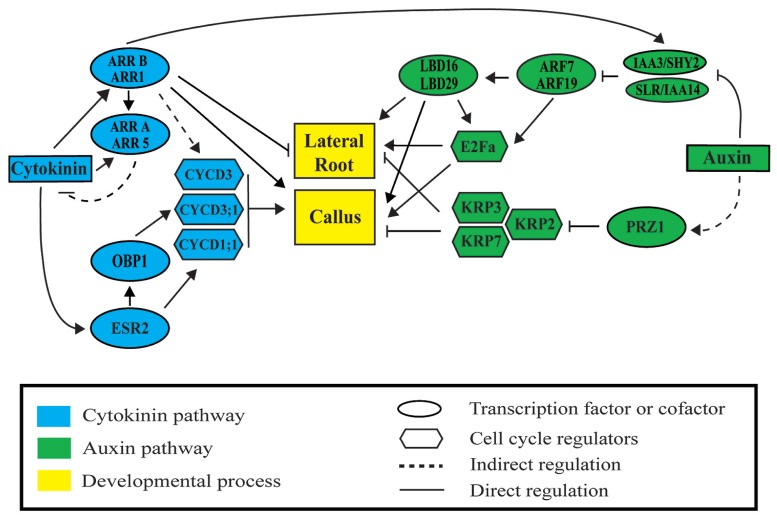
**Auxin and cytokinin signaling modules regulate lateral root and callus formation.** Auxin activates lateral root and callus initiation through derepression of AUXIN RESPONSIVE FACTORS (ARF) by INDOLE-3-ACETIC ACID (AUX/IAA) proteins. LATERAL BOUNDARIES DOMAIN (LBD) factors are then activated to initiate LR or callus formation. Cytokinin represses lateral root initiation and activates callus formation through type B *ARABIDOPSIS* RESPONSE REGULATORS (ARR). Auxin and cytokinin regulate cell division through ARF-LBD factors, PROPORZ1 (PRZ1), ENHANCED SHOOT REGENERATION 2 (ESR2), OBF BINDING PROTEIN 1 (OBP1), and type B ARRs. Upon activation, downstream cyclin-dependent protein kinases D (CYCD), the transcription factor E2Fa, and the INHIBITORY PROTEIN (KIP)-RELATED PROTEINS (KRP) regulate progression through the cell cycle during lateral root and callus formation.

Lateral root (LR) and callus formation is regulated by the hormones auxin, cytokinin and their downstream signaling pathways (**Figure 2**). Different concentrations of these hormones regulate the balance between cell proliferation and differentiation (Dello Ioio et al., 2008). Auxin and cytokinin are components of CIM and at the used ratio (high auxin and low cytokinin levels) they maintain cells in a proliferative state. Subsequently, callus exposed to different ratios of auxin–cytokinin can be induced to form adventitious roots or shoots (Skoog and Miller, 1957). Likewise, a whole plant can be regenerated from multiple tissues following a temporal series of hormonal treatments. Auxin and cytokinin are also involved in LR formation. LR formation requires the function of both hormones in the early differentiation zone of the root described as the developmental window. This is the region of the root where LR initiation takes place (Bielach et al., 2012; Van Norman et al., 2013). Auxin function in LR founder cells is essential to promote LR initiation (Casimiro et al., 2001; Dubrovsky et al., 2008) while cytokinin inhibition prevents ectopic LR initiation in pericycle cells (Bielach et al., 2012). Thus, cytokinin regulation occurs simultaneously to LR initiation but in different cell types.

As auxin and cytokinin downstream pathways operate both during post-embryonic and *de novo* organogenesis, common transcriptional regulation is expected. A meta-analysis of genome-wide transcriptomic datasets, which profiled (1) callus at different time points during CIM incubation (Xu et al., 2012) and (2) LR initiation in the LR inducible system (Vanneste et al., 2005; De Smet et al., 2008), shows common regulation of genes (Motte et al., 2014). 847 genes upregulated in callus were also upregulated in dividing pericycle cells (out of 1109 genes), and 643 genes activated in callus were also genes identified as LR initiation genes (out of 913). Some of these genes have been described in both developmental programs. Next, we compare auxin and cytokinin pathways in LR and callus formation highlighting common regulators of both developmental processes.

## AUXIN REGULATES LATERAL ROOT INITIATION AND CALLUS FORMATION

Auxin function in LR formation requires its biosynthesis, transport, and signaling. The TRYPTOPHAN AMINOTRANSFERASE OF *ARABIDOPSIS* 1 (TAA1) acts in the indole-3-pyruvic acid branch of the auxin biosynthetic pathway. Loss of function mutants of *TAA1* (*tir2*) have impaired LR formation that can be rescue by treatment with auxin and the auxin biosynthetic intermediate like indole-3-pyruvic acid (Stepanova et al., 2008; Yamada et al., 2009). Exogenous auxin treatment as well as its normal transport and accumulation during root development can induce LR formation (Fukaki et al., 2002; Himanen et al., 2002; Laskowski et al., 2008). Accordingly, auxin transport inhibitor *N*-1-naphthylphthalamic acid blocks LR development before the first asymmetric division of pericycle cells (Casimiro et al., 2001). Thus, both auxin and its transport are necessary for LR initiation and organogenesis. Auxin is also an essential component in CIM and required for callus formation. The synthetic auxin 2,4-D, even without cytokinin, produces activation of cell division leading to formation of proliferative masses of cells (Atta et al., 2009).

Auxin accumulation in xylem pole pericycle may trigger specification of LR founder cells (Dubrovsky et al., 2008). The PIN3 auxin efflux carrier has been described to participate in auxin accumulation in LR founder cells from the endodermis through a reflux mechanism. Missing this auxin transporter causes defects in subsequent LR initiation (Marhavy et al., 2013). Once specified, LR founder cells normally undergo divisions to initiate a new LR. These divisions are asymmetric and very likely also require auxin accumulation as indicated by enhanced auxin transcriptional response and expression of auxin carriers (Malamy and Benfey, 1997; Benkova et al., 2003; De Smet, 2012; Peret et al., 2013). Thus, LR formation from LR founder cells has been correlated with expression of the auxin influx carrier AUX1. AUX1 activity may result in auxin accumulation necessary for development of new LRs (De Smet et al., 2007a). Auxin transport has not been described to occur during callus formation. However, callus cells organize in a similar fashion as a root meristem and auxin transport is required for root meristem organization and activity (Grieneisen et al., 2007; Cruz-Ramirez et al., 2012). Thus, it is tempting to speculate if callus organization, might also use auxin transport.

Lateral root initiation requires the asymmetric cell division mediated by ALF4. *alf4* loss of function mutant is epistatic to auxin signaling (Celenza et al., 1995) which indicates that LR initiation mediated by auxin requires this regulator. This mutant has also reduced callus formation (DiDonato et al., 2004; Sugimoto et al., 2010). Callus formation might, therefore, require the same set of regulators to interact with auxin signaling.

Auxin signaling is delivered by AUXIN RESPONSE FACTOR (ARF) proteins, which function transcriptionally as activators or repressors (Ulmasov et al., 1999; Tiwari et al., 2003), and AUXIN/INDOLE-3-ACETIC ACID (AUX/IAA) proteins. AUX/IAA proteins, in absence of auxin, repress ARF activity by heterodimerization, while in the presence of auxin AUX/IAA are degraded (Zenser et al., 2001). ARFs can then act to regulate genes in the auxin pathway. Auxin signaling modules are necessary for LR organogenesis and recent evidence shows that they appear to be also necessary for callus formation (Fan et al., 2012). Dominant mutants of AUX/IAAs which repress auxin signaling show impairment in LR initiation. The SOLITARY ROOT/IAA14 dominant mutant (*slr-1/iaa14)* shows a strong phenotype with no LR formation. This mutation blocks asymmetric cell divisions of LR founder cells during LR initiation (Fukaki et al., 2002; Vanneste et al., 2005). A gain-of-function mutant of IAA28 suppresses the auxin response which leads to LR founder cell activation. Subsequent decrease in LR initiation might occur through ARF5 to ARF8, and ARF19 signaling (De Rybel et al., 2010). Furthermore, the double mutant *arf7 arf19* shows a similar phenotype to *slr-1/iaa14,* with no LR initiation as well as others auxin related phenotypes. ARF7 and ARF19 develop a redundant function and consequently the single mutants do not have obvious phenotypes (Okushima et al., 2005). Interestingly, *arf7 arf19* double mutant also shows impaired callus formation (Fan et al., 2012). Although ARFs are involved in callus formation, there is no evidence for AUX/IAA proteins. Future experiments might show their implication.

AUXIN RESPONSE FACTORs regulates LATERAL ORGAN BOUNDARIES-DOMAIN/ASYMMETRIC LEAVES2-LIKE-(LBD/ASL) transcription factors during LR organogenesis. It has been described that ARFs primarily regulate LR initiation via direct activation of *LBD/ASL* genes (**Figure 2**; Okushima et al., 2007). Thus, overexpression of *LBD16/ASL18* and *LBD29/ASL16* can partially restore the *arf7 arf19* phenotype, which rescues LR initiation. LBD16/ASL18 has been described to be specifically expressed in pairs of xylem pole pericycle before the first asymmetric cell division. As LBD16/ASL18 functions are redundant with other LBDs/ASLs a dominant repressor form of this protein, LBD16-SRDX, was used. LBD16-SRDX blocks the first asymmetric cell division necessary for LR initiation (Goh et al., 2012a). A recent study in callus formation has demonstrated that four *LBDs/ASL* genes (*LBD16/ASL18, LBD17/ASL15, LBD18/ASL20, and LBD29/ASL16*) are rapidly activated by CIM. Furthermore, ectopic expression of these *LBDs/ASLs* can induce callus formation without plant hormones treatment; whereas T-DNA insertion mutants (*lbd16-2* and *lbd18-1*), inhibit callus formation. These four LBD/ASL factors are downstream of ARF7 and ARF19 (Fan et al., 2012). This indicates that LBDs/ASLs are key regulators of callus formation. This regulation might occur through activation of asymmetric divisions, similarly to the mechanism described for LR initiation. In a nutshell, as expression of LBD16/ASL18 and LBD29/ASL16 is also regulated by SLR/IAA14-ARF7-ARF19 signaling module (Goh et al., 2012a), it appears that the same set of regulators are require for both LR initiation and callus formation.

## CYTOKININ INHIBITS LATERAL ROOT INITIATION AND IT IS REQUIRED FOR CALLUS FORMATION

Cytokinin is an important component of CIM. Skoog and Miller (1957) described that cytokinin is essential for callus formation. They describe the cytokinin effects while they were assaying different auxin–cytokinin ratios in the formation of callus. CIM has low cytokinin concentration, which is presumably required to maintain proliferation when high auxin concentrations are supplied. Some authors suggest that endogenous levels of cytokinin in tissue explants are sufficient to preserve growth (del Pozo et al., 2005; Gordon et al., 2007). In fact, it has been described that callus can be produced without cytokinin in the medium (Atta et al., 2009).

The role of cytokinin during post-embryonic development has been described as that of a LR organogenesis inhibitor with opposed effect to auxin (Li et al., 2006). High levels of cytokinin in LR founder cells are capable of limiting its subsequent development into LRs (Laplaze et al., 2007). In agreement with this observation, reduced levels of cytokinin, achieved through transgenic plants overexpressing CYTOKININ OXIDASE/DEHYDROGENASE (CKX), result in increased numbers of LRs (Werner et al., 2003). Exogenous application of cytokinin during LR initiation blocks pericycle founder cell divisions and this phenotype cannot be rescued by exogenous auxin treatment (Li et al., 2006). In addition, LR founder cells and xylem pole pericycle cells near developing primordia are highly sensitive to cytokinin signaling while LR primordia are less sensitive (Laplaze et al., 2007; Bielach et al., 2012). Cytokinin signaling appears to prevent excessive nearness among LRs. In agreement with this observation, TCS cytokinin transcriptional reporter is expressed in pericycle cells of the developmental window and between two existing LR primordia (Bielach et al., 2012). The function of cytokinin as LR formation inhibitor has been also observed during *de novo* organogenesis from callus or explants. High concentration of cytokinin reduces root formation to promote shoot differentiation. Cytokinin has been described to inhibit root identity genes when callus are transferred to shoot induced medium (SIM; Atta et al., 2009). In contrast, roots cultured in medium which only contains auxin differentiate LRs (De Smet et al., 2007b; Atta et al., 2009). In CIM, the combined action of auxin and cytokinin results in formation of callus which expresses root meristematic genes, even when high levels of cytokinin are used (Sugimoto et al., 2010). Auxin in CIM has been describe to be responsible of proliferation of xylem pole pericycle cells leading to generation of callus (Atta et al., 2009), while the inhibitory effects of cytokinin would prevent differentiation of proliferating cells into LRs.

Cytokinin response can be mediated by the *ARABIDOPSIS* RESPONSE REGULATORS (ARRs). These proteins can be classified in two groups, type A and type B. Type A are rapidly upregulated by exogenous cytokinin and repress cytokinin signaling. In *Arabidopsis*, there are 10 members of the type A group (ARR3 to ARR9 and ARR15 to ARR17; D’Agostino et al., 2000). Type B ARRs (ARR1, ARR2, ARR10 to ARR14, ARR18 to ARR21, and ARR23) are transcription factors that mediate cytokinin response through activation of gene expression (Sakai et al., 2001; Su and Zhang, 2014). Type A ARRs are transcriptionally induced by type B ARRs. Type A ARR15 and ARR5 are upregulated on SIM. Tissue explants directly cultured on SIM show upregulation of *ARR5*, while *ARR15* activation requires previous callus formation in CIM. It has been hypothesized that *ARR15* is normally blocked by a repressor, which, in turn, would be repressed in CIM (Che et al., 2008). It is usual to find redundancy between members of the ARR family (Sakai et al., 2001; Mason et al., 2005). The double loss of function mutant of *ARR8* and *ARR9* genes shows reduced number of LRs. This indicates that ARR8 and ARR9 are repressors of cytokinin signaling during LR formation. Similarly, the hextuple mutant *arr3, 4, 5, 6, 8, and 9* presents increased cytokinin sensitivity during LR formation, and the phenotype aggravates when compared with fewer order mutants (To et al., 2004). Type A ARRs act both during LR initiation and callus formation; however, only ARR5 has been found to be a common regulator of both processes.

Type B ARR1 is involved in callus formation. Plants overexpressing *ARR1* and *arr1-1* mutants are respectively more and less sensitive to cytokinin treatments than the wild-type. Thus, callus derived from *ARR1* overexpressing plants produces more shoots upon cytokinin treatment while *arr1-1* derived callus produced less (Sakai et al., 2001). During post-embryonic root development, double loss of function mutant *arr1 arr11* produces abnormal positioning of LR primordia. This would be related with the mentioned role of cytokinin in preventing LR initiation near existing LR primordia (**Figure 2**; Bielach et al., 2012). ARR1 functions as repressor of root formation both during plant regeneration and post-embryonic root development. In addition, ARR1 is connected with auxin signaling as ARR1 directly activates *SHY2/IAA3*. *SHY2/IAA3* is thus activated by cytokinin and, in addition, is required for repression of auxin transport and signaling (Dello Ioio et al., 2008). Intriguingly, the auxin resistant form of this protein (*shy2-101)* has more free auxin and shows excessive proliferation of pericycle cells in mature parts of the root. These dividing pericycle cells do not differentiate into LRs (Goh et al., 2012b). This phenotype resembles callus initiation. Future research appears to be required to dissect the role of this regulator during auxin and cytokinin interaction.

## COMMON CELL CYCLE REGULATION DURING LATERAL ROOT AND CALLUS FORMATION

The signaling mechanisms mediated by hormones during callus formation may converge in regulation of the cell cycle. In *Arabidopsis alf4-1* mutants, cell division is almost blocked (DiDonato et al., 2004; Sugimoto et al., 2010). It has been suggested that the *ALF4*-encoded protein, which is evolutionary conserved among taxa, maintains pericycle cells in a mitotically competent state. This indicates that progression through the cell cycle appears to be required for organogenesis under endogenous and exogenous cues such as hormonal treatments. Future studies might address the exact mode of action of ALF4 in regulation of asymmetric cell division.

The *Arabidopsis* transcription factors LBD16/ASL18, LBD29/-ASL16, and LBD18/ASL20 are important constituents of the auxin signaling pathway operating downstream of ARF7 and ARF19. LBD18/ASL20 and LBD33/ASL24 mediate LR organogenesis through formation of protein dimers which bind to the promoter region of *E2Fa* to activate its transcription (Berckmans et al., 2011). E2Fa is one of the six E2F transcription factors in *Arabidopsis* which through dimerization with a *DIMERIZATION PARTNER* (*DP*) promotes transcriptional activation of the cell cycle (De Veylder et al., 2002; Sozzani et al., 2006). LBD16/ASL18, LBD29/ASL16, and LBD18/ASL20 are also involved in callus formation. These *LBD* genes or their homolog LBD17/ASL15 are rapidly induced by CIM in multiple organs (Fan et al., 2012). Ectopic expression of any of these *LBD* genes in *Arabidopsis* is sufficient to trigger callus formation without supplementation of exogenous hormones. This suggests that *LBD* genes might induce callus formation through activation of *E2Fa* (**Figure 2**). However, there is no evidence that *E2Fa* overexpression induces callus formation even when co-expressed with its dimerizing partner *DPA* (De Veylder et al., 2002). Thus ectopic expression of *E2Fa* and *DPA* during LR development is not sufficient to form new LRs. However, pericycle cells undergo several rounds of proliferative cell division giving rise to stretches of divided pericycle cells typical of LR initiation (Vanneste et al., 2005; De Smet et al., 2010). Likely, callus formation through E2Fa/DPA dimers requires other LBD targets, although it is unknown if they might be cell cycle regulators.

Interestingly, cell division genes are early up-regulated after callus induction (Atta et al., 2009; Xu et al., 2012). Their transcriptional activation presumably results in activation of cell cycle and the formation of proliferating masses of cells, which are found in calli. Among these cell division genes there are cell cycle regulators, genes encoding chromatin structural proteins and proteins related to DNA synthesis machinery such as *CYCA2;4*, *CYCB1;1*, *CYCA1;1*, *CYCB2;4*, *CYCB1;3*, *CYCD3;3*, the cyclin-dependent kinase (CDK) *CKS2* or the cell division protein *APC6* (Xu et al., 2012). Several of these genes also have a function during root development. Mutants of A2-type cyclins (*cyca2s*) display reduced LR density and deviations in LR primordium patterning (Vanneste et al., 2011).

Expression of *CYCD3* is induced by cytokinin as a potential target of type-B ARRs. Analyses of ARR type B mutants reveal progressively decreased sensitivity to cytokinin, including effects on root elongation, LR formation and callus induction (Mason et al., 2005). Plants overexpressing *CYCD3* render callus formation in CIM without cytokinin, suggesting that *CYCD3* is a key target of cytokinin in regulation of callus formation (Riou-Khamlichi et al., 1999). Another member of the CYCD protein family, CYCD1;1, is also regulated downstream of cytokinin during callus formation. Expression of *CYCD1;1* is controlled by the transcription factor ENHANCED SHOOT REGENERATION 2 (ESR2). Overexpression of *ESR2* induces callus without hormonal treatment and shows elevated cytokinin response (**Figure 2**; Banno et al., 2001; Ikeda et al., 2006). ESR2 is also activating the expression of OBF BINDING PROTEIN 1 (OBP1). OBP1 promotes cell cycle reentry by shortening the duration of the G1 phase. In addition, it regulates expression of the cell cycle-associated genes *CYCD3;3* and *DOF2;3* through direct binding to their promoters (Skirycz et al., 2008).

Cell cycle repressors may be down regulated upon hormonal treatment. The cell cycle repressors INTERACTOR OF CYCLIN-DEPENDENT KINASE and KINASE INHIBITORY PROTEIN (KIP)-RELATED PROTEIN (ICK/KRP) are down regulated after auxin treatment, being the transcriptional adaptor protein PROPORZ1 (PRZ1) responsible of this repression (**Figure 2**; Anzola et al., 2010). PRZ1 is involved in the modulation of histone modifications at the KRP loci in response to auxin. The *prz1* mutant presents hyper proliferative growth and de-regulation of *KRP* expression. KRP silencer lines phenocopy the *prz1* phenotype, whereas *KRP* overexpression suppresses the mutant phenotype. However, how auxin modulates *PRZ1* expression remains unknown. KRPs proteins also prevent auxin-mediated LR initiation. Mutants of *KRP2*, *krp2*, and its overexpression display increased and reduced LR density, respectively, which occurs through the inactivation of CYCD2;1 (Himanen et al., 2002; Ren et al., 2008; Sanz et al., 2011).

Activation of single core cell cycle regulators, such as cyclins (CYCs) or CDKs is not sufficient to induce callus or LR development (Riou-Khamlichi et al., 1999; Cockcroft et al., 2000; Dewitte et al., 2003; Vanneste et al., 2005). However, activation of the basic cell cycle machinery in combination with auxin treatment enhances capacity of pericycle cells to form new LRs (De Smet et al., 2010). This regulatory mechanism appears to be similar to that operating during callus formation as overexpression of *CYCD3* is able to form callus in auxin containing medium (Riou-Khamlichi et al., 1999). Callus and LR formation appear to share common cell cycle regulators. In turn, these regulators are controlled by the auxin and cytokinin signaling pathways. Future work might address crosstalk relationships between these two hormone signaling pathways to regulate the cell cycle during LR formation or *de novo* organogenesis via callus formation.

## EPIGENETIC REGULATION INTEGRATES DEVELOPMENTAL PROGRAMS DURING LR ORGANOGENESIS AND CALLUS FORMATION

Callus formation requires dramatic changes in both cell identities and cell growth patterns. These changes have been shown to be accompanied by activation or repression of numerous genes across the genome (Atta et al., 2009; Sugimoto et al., 2010). It seems unlikely that these genome-wide changes of gene expression are only achieved through the spatial and temporal regulation delivered by transcription factors. Plant epigenetic pathways, which are known to globally influence gene expression, may also participate in gene expression regulation during callus formation (Li et al., 2011; Xu and Huang, 2014).

In both animals and plants the Polycomb group (PcG) proteins act in an evolutionarily conserved epigenetic pathway that regulates chromatin structure. PcG proteins repress many developmentally important genes through modification of histones. PcG proteins can form at least two multiprotein complexes: the Polycomb repressive complexes 1 and 2 (PRC1 and PRC2). In plants, the major function of PRC2 is to trimethylate lysine 27 on histone H3 (H3K27me3); while PRC1 recognizes the H3K27me3 marker and mono-ubiquitinates histone H2Aub (Schatlowski et al., 2008; Bratzel et al., 2010; Molitor and Shen, 2013). Both epigenetics marks contribute to stabilize the repression of embryonic and meristematic programs in differentiating organs. The *Arabidopsis* proteins CURLY LEAF (CLF), SWINGER (SWN), VERNALIZATION (VRN2) and EMBRYONIC FLOWER2 (EMF2) participate as core components of PRC2 (Goodrich et al., 1997; Gendall et al., 2001; Yoshida et al., 2001; Chanvivattana et al., 2004). Double mutants of these homologs exhibit spontaneous callus generation soon after germination (Chanvivattana et al., 2004; Schubert et al., 2005). Similarly, the mutant of FERTILIZATION INDEPENDENT ENDOSPERM (FIE), another component of PRC2, also forms spontaneous callus formation (Bouyer et al., 2011). Double mutants *clf-50 swn-1* fail to form callus from leaf explants but not from root explants, which indicates that the PRC2 components, CLF and SWN, repress shoot fate genes during callus formation (He et al., 2012). Thus it appears that although calli formed form aerial organs follow a root development pathway (Sugimoto et al., 2010), shoot fate needs to be repressed during callus induction.

*Arabidopsis AtBM1A* and *AtBM1B* genes are the homologs of mammalian *PRC1* gene (Sanchez-Pulido et al., 2008). The aerial parts of the double mutant *Atbm1aAtbm1b* are unable to maintain differentiation and form both embryo- and callus-like structures. In addition, root architecture of this mutant during post-embryonic development is altered showing a phenotype reminiscent of other epigenetic regulators (Ogas et al., 1997; Bratzel et al., 2010). In *Arabidopsis* PRC1 mutants, several embryonic regulators are overexpressed such as LEAFYCOTYLEDON 1 (LEC1), LEAFYCOTYLEDON2 (LEC2), AGAMUS-LIKE 15 (AGL15), or BABY BOOM (BBM). As over expression of embryonic fate regulators results in formation of ectopic embryos (Boutilier et al., 2002; Tsuwamoto et al., 2010), it is possible that overexpression of *BBM* or other embryonic fate regulators could lead to formation of embryonic structures in ectopic callus of *Atbm1aAtbm1b* mutants. Other genes would then be necessary in *Atbm1aAtbm1b* to form no-embryonic callus.

The *Arabidopsis* CHD3/4-related regulator PICKLE (PKL) also plays a significant role in transcriptional repression of cell identity genes and *pkl* mutants spontaneously develop callus after germination (Ogas et al., 1997, 1999). A new mutant allele of *PKL* gene, called *cytokinin-hypersensitive1*, rapidly produces calli in response to lower levels of cytokinins (Furuta et al., 2011). Interestingly, treatment of explants with trichostatin A (TSA), an inhibitor of histone deacetylase, leads to similar effects as cytokinin in callus formation (Furuta et al., 2011). This indicates that chromatin remodeling and histone deacetylations are intimately related to cytokinin activity in callus formation. PKL is also implicated in LR development. *suppressor of iaa14/slr2* (*ssl2*) mutant turns out to be another mutant allele of *PKL*. In addition, treatments with TSA partially suppress the phenotype of *slr1* (Fukaki et al., 2006).Therefore, PKL/SSL2 is required for the SLR/IAA14-mediated suppression of LR initiation and to negatively regulate auxin-mediated LR formation.

Recent studies have shown that some chromatin modifiers may interact directly with transcription factors to change the epigenetic status and expression of specific target genes (Fukaki et al., 2006; Zhou et al., 2013). It could be interesting to explore if the same set of transcription factors would interact with chromatin modifiers both during LR and callus formation. Moreover, it could be of great interest to dissect the role auxin and cytokinin signaling pathways in these specific interactions.

## CONCLUDING REMARKS

Over the last decades, developmental and molecular biologists have dissected the pathways and regulators involved in cell fate reprogramming and organogenesis under post-embryonic developmental programs. The use of similar approaches to analyze callus formation during *de novo* organogenesis has provided striking insight into the mechanistic regulation of this process. Unifying principles and paradigms of common regulation start to emerge: developmental regulators of LR initiation are central to plant regeneration through callus formation. Thus, it appears sensible to further study the function of known post-embryonic developmental pathways during callus formation, as this research could provide more in depth understanding of the mechanisms that operate at the molecular level. The role of cross-talk between the hormones auxin and cytokinin or the mechanistic regulation of gene expression downstream of epigenetic regulation appear as particularly intriguing, and yet to explore during callus formation.

Developmental biology can also be benefited from the understanding of processes driven by exogenous cues (hormonal supplementation) at the molecular level. Example of this is the LR inducible system, which has been useful to find novel regulators of LR formation but also of stem cell niche function (De Smet et al., 2008). Finally, integration of knowledge into functional predictive models and the use of system biology approaches will help to unravel global regulatory mechanisms involved in organogenesis. Understanding organogenesis in the context of global regulation and signaling driven by endogenous cues and hormonal treatments could presuppose strategic advantage in order to develop new biotechnological tools.

## Conflict of Interest Statement

The authors declare that the research was conducted in the absence of any commercial or financial relationships that could be construed as a potential conflict of interest.
